# Detection of Snake Venom in Post-Antivenom Samples by Dissociation Treatment Followed by Enzyme Immunoassay

**DOI:** 10.3390/toxins8050130

**Published:** 2016-04-28

**Authors:** Kalana P. Maduwage, Margaret A. O’Leary, Anjana Silva, Geoffrey K. Isbister

**Affiliations:** 1 Clinical Toxicology Research Group, University of Newcastle, Newcastle 2298, Australia; kalanapm@gmail.com (K.P.M.); margaret.Oleary@newcastle.edu.au (M.A.O.); 2 South Asian Clinical Toxicology Research Collaboration, University of Peradeniya, Peradeniya, Sri Lanka; 3 Department of Biochemistry, Faculty of Medicine, University of Peradeniya, Peradeniya, Sri Lanka; 4 Monash Venom Group, Monash University, Melbourne 3168, Australia; anjana.silva@monash.edu

**Keywords:** venom, antivenom, dissociation, enzyme immunoassay, venom detection, snakebite

## Abstract

Venom detection is crucial for confirmation of envenomation and snake type in snake-bite patients. Enzyme immunoassay (EIA) is used to detect venom, but antivenom in samples prevents venom detection. We aimed to detect snake venom in post-antivenom samples after dissociating venom-antivenom complexes with glycine-HCl (pH 2.2) and heating for 30 min at 950 °C. Serum samples underwent dissociation treatment and then Russell’s viper venom or Australian elapid venom measured by EIA. In confirmed Russell’s viper bites with venom detected pre-antivenom (positive controls), no venom was detected in untreated post-antivenom samples, but was after dissociation treatment. In 104 non-envenomed patients (negative controls), no venom was detected after dissociation treatment. In suspected Russell’s viper bites, ten patients with no pre-antivenom samples had venom detected in post-antivenom samples after dissociation treatment. In 20 patients with no venom detected pre-antivenom, 13 had venom detected post-antivenom after dissociation treatment. In another 85 suspected Russell’s viper bites with no venom detected pre-antivenom, 50 had venom detected after dissociation treatment. Dissociation treatment was also successful for Australian snake envenomation including taipan, mulga, tiger snake and brown snake. Snake venom can be detected by EIA in post-antivenom samples after dissociation treatment allowing confirmation of diagnosis of envenomation post-antivenom.

## 1. Introduction

Snake envenomation remains a neglected tropical disease with large numbers of cases in resource poor countries, many without antivenom [1]. In some cases snake envenomation syndromes are poorly defined and basic clinical research is required to better define human envenomation and the effect of interventions such as antivenom. Enzyme immunoassay (EIA) has been used to detect snake venom in envenomed patients’ samples for the last four decades [2]. Detection and identification of snake venom is particularly crucial in clinical trials [3,4,5] as well as in prospective studies of definite snakebite cases to correctly define the effects of snake envenomation and the effect of different interventions. This avoids the need to collect and identify snakes that bite patients, which is only possible in a proportion of cases. Currently, this approach depends on the availability of a pre-antivenom serum sample to confirm snake venom by EIA. Lack of a pre-antivenom serum sample can lead to the exclusion of patients from some clinical studies. In addition, confirmation of envenomation in forensic cases is important [6]. However, in such cases after antivenom is given there is currently no method to detect venom. Therefore, detection of snake venom in post-antivenom samples may increase the number of definite cases in clinical studies and confirmation of snake envenomation in forensic samples.

Specific venom EIAs rely on snake venom antigens in blood samples binding to anti-snake toxin antibodies bound to a microplate. These venom antigens are then detected by labelled anti-snake antibodies, a technique referred to as a sandwich EIA [5,7]. A recent study has shown that this venom specific EIA can also detect venom which is bound to antivenom [8,9]. However, this does not occur if there is excess antivenom present, which is usually the case after the administration of therapeutic antivenom. To be able to measure venom in the presence of excess antivenom the venom-antivenom complex needs to be dissociated and antivenom removed (destroyed) so that venom can then be detected.

In this study, we investigate a method to dissociate venom-antivenom complexes in post-antivenom samples, enabling detection of the venom by EIA. The method is tested in three series of post-antivenom samples from envenomed patients.

## 2. Results

### 2.1. Demonstration of Venom-Antivenom Dissociation

**Figure 1:**
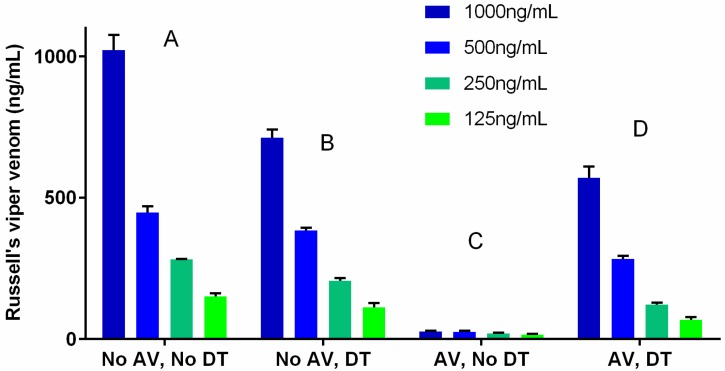
Effect of dissociation treatment (DT; treatment with glycine-HCl buffer of pH 2.2 and heated at 95 °C for 30 min) on the detection of free venom at four concentrations (mean ± SEM) of Russell’s viper venom (RVV; ng/mL), with and without antivenom (AV; 10 mg/mL). The four sections of the graph represent solutions with no antivenom and no DT (Panel **A**), no antivenom with DT (Panel **B**), with antivenom but no DT (Panel **C**) and with antivenom and DT (Panel **D**).

An initial trial of dissociation conditions, using buffers of pH 3.6 and 2.2, and temperatures of 37 °C or 95 °C, found that the most extreme conditions were more successful in recovering measureable venom from venom-antivenom complexes, without significant loss of free venom due to the harsh conditions. Solutions of Russell’s viper (*Daboia russelii*) venom (RVV) at concentrations of 1000 ng/mL, 500 ng/mL, 250 ng/mL and 125 ng/mL with and without antivenom were treated with glycine-hydrochloric acid (HCl) buffer of pH 2.2 and heated at 95 °C for 30 min (dissociation treatment). The recovery of free venom in solutions without antivenom ranged from 70% to 86% (Figure 1; ratio of panel B to panel A). When the same solutions had antivenom (10 mg/mL) added and treated the same way, the recovery of free venom from venom-antivenom complexes ranged from 43% to 65% (Figure 1; ratio of panel D to panel A). From this, the limit of detection of RVV in samples with dissociation treatment was estimated to be 50 ng/mL. Measurement of antivenom in treated samples gave absorbance values not significantly different from samples to which no antivenom had been added, showing that antivenom (antibodies) is destroyed by the dissociation conditions.

**Figure 2:**
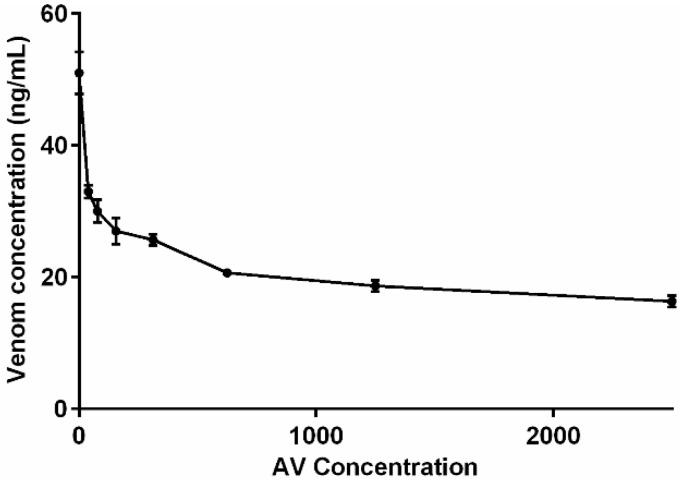
Plot of the measured venom concentration (mean ± SEM) versus antivenom concentration for venom-antivenom mixtures initially containing 50 ng/mL brown snake (*Pseudonaja textilis*) venom, after dissociation treatment.

Solutions of brown snake (*Pseudonaja textilis*) venom at a concentration of 50 ng/mL were prepared with increasing concentrations of brown snake antivenom (0 to 2500 mU/mL), and subjected to the dissociation treatment. Recovery of venom was near 100% in samples with no antivenom, declining to <40% at very high concentrations of antivenom (Figure 2).

In some cases of envenomation by Australian snakes, samples after dissociation treatment were measured for venom with and without the addition of antivenom. In the former case, venom was detected and then was not detectable after the addition of antivenom (data not shown). This ensures that the absorbance is not due to background, and is important for testing cases with low concentrations of venom near the limit of detection.

### 2.2. Detection of Venom after Venom-Antivenom Dissociation in Patient Samples

The venom-antivenom dissociation treatment was used in thawed serum samples collected from two series of Sri Lankan Russell’s viper bites (Series 1 and 2) and one series of Australian snake bites (Series 3).

**Figure 3:**
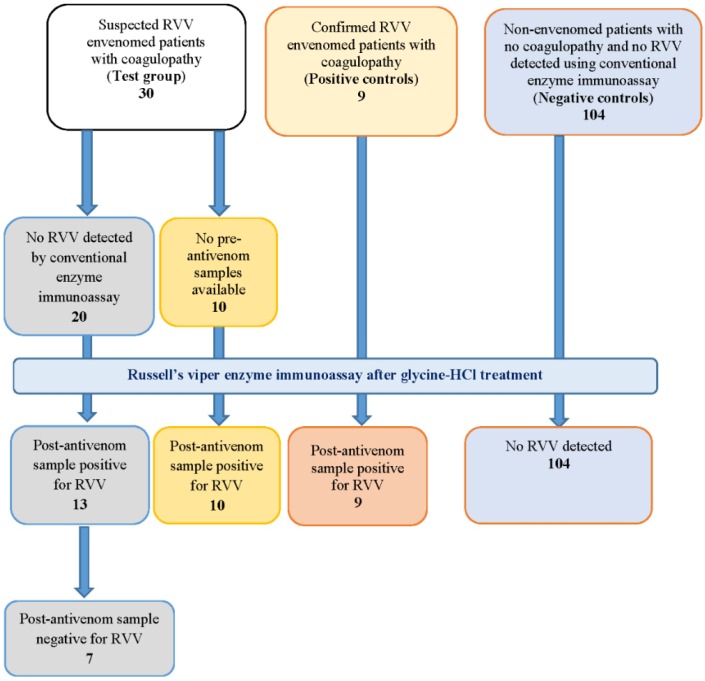
Detection of Russell’s viper venom (RVV) by enzyme immunoassay after dissociation treatment of positive and negative controls, and patients with suspected Russell’s viper envenomation.

Series 1 included 143 patients recruited to a prospective cohort study of snakebites in north central Sri Lanka. The series consisted of 9 patients with venom detected pre-antivenom (positive controls), 104 non-envenomed patients (negative controls) and 30 patients with suspected Russell’s viper bites who had either no pre-antivenom samples (10) or no venom detected in the pre-antivenom samples (20) (Figure 3). Venom was detected in all nine positive controls (False negative rate 0%; 95% Confidence intervals (CI): 0% to 37%) and was not detected in the 104 negative controls (False positive rate 0%; 95% CI: 0% to 4%). The latter demonstrates that the dissociation treatment does not produce false positives by measuring venom in samples without venom present. In all ten patients with no pre-antivenom samples venom was detected post-antivenom after dissociation treatment. Twenty patients had no venom detectable in their pre-antivenom samples, with or without dissociation treatment. In 13 of these 20, venom was detected in post-antivenom samples after dissociation treatment. There were seven patients for which venom could not be detected at all in any post-antivenom sample even after dissociation treatment. No particular bite site, time post-bite or number of antivenom doses given was associated with these seven patients.

Series 2 was a prospective cohort study of Russell’s viper bites recruited to a study of neurotoxicity [10]. In this series, 85 of 216 patients had no venom detectable in their admission or pre-antivenom samples, and no snake had been properly identified. After dissociation treatment, at least one sample from 50 of these patients revealed the presence of RVV, enabling the inclusion of these additional patients in the study.

**Figure 4:**
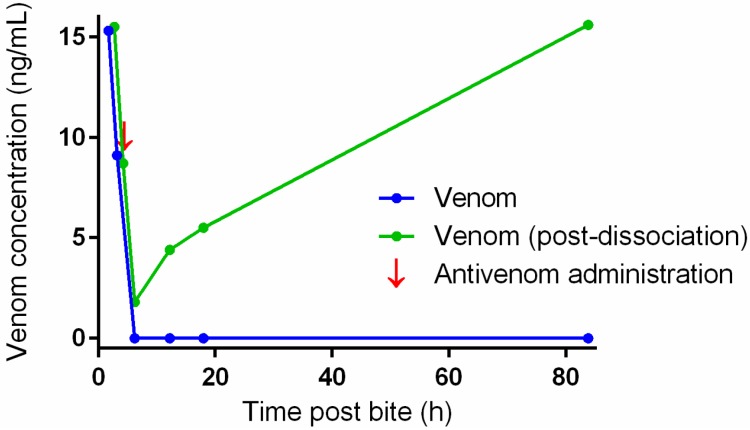
Plots of venom concentrations versus time post-bite before (filled circles and line) and after (filled squared and dashed line) dissociation treatment for a case of taipan envenomation. The arrow marks the time of the administration of antivenom.

Series 3 included snake envenomation cases from the Australian Snakebite Project (ASP) which is a prospective study of snakebites across all Australia where serum samples are collected and frozen from recruited patients. Serum samples were subjected to dissociation treatment to confirm the type of snake in patients where pre-antivenom samples were not available. If no venom from the suspected snake type was detected after dissociation treatment, then the next most likely venom assay was done, based on geography and clinical effects. There were ten patients with suspected taipan (*Oxyuranus scutellatus*) envenomation. Four patients had taipan venom detected pre-antivenom and also after dissociation treatment in post-antivenom samples (positive controls; Figure 4). Six had no pre-antivenom samples of which four had taipan venom detected after dissociation treatment post-antivenom. In the two remaining cases no taipan venom was detected after dissociation treatment, but brown snake venom was detected in one of these being the next most likely snake in this region. There were four suspected mulga snake (*Pseudechis australis*) envenomations with no pre-antivenom samples. In two patients mulga snake venom was detected after dissociation treatment. In the remaining two, mulga snake venom was not detected, but in one brown snake venom was detected, again being the next most likely snake. Two cases of tiger snake envenoming were confirmed by subjecting post-antivenom samples to the dissociation treatment.

## 3. Discussion

This study shows that venom can be detected in post-antivenom samples by dissociating venom and antivenom complexes with acid and heat. In three series of patients in which no pre-antivenom samples were available or in which venom could not be detected in pre-antivenom samples, venom was detected post-antivenom using this dissociation method. This allows the positive identification of cases for inclusion in research studies, as in series 2, or forensic identification of cases where only post-antivenom samples may be available. The measurement of venom in post-antivenom samples was shown not to be due to artefact by using negative control samples, and by the addition of antivenom to samples after dissociation, and re-measuring.

Dissociation of immune complexes with the aim of enabling antigen identification has been done with a number of other substances. Heating samples with ethylenediaminetetraacetic acid (EDTA) was found to improve the detection of *Histoplasma* and *Coccidioides* antigens [11,12], while sodium dodecyl sulfate (SDS) has been used in studies of autoimmune disease [13]. Heating in an acid such as HCl [14], acetic acid [15] or acid glycine [16,17] is a common approach for dissociating antigens and antibodies. Gustaw *et al*. used this technique to unmask anti-amyloid-β antibodies previously undetectable because of the presence of excess antigen [17]. Of these reagents, we found that glycine-HCl with heat gave the best recovery of venom from venom-antivenom complexes.

Antivenom was destroyed by heating (to 95 °C) in glycine-HCl and so was no longer detectable using a venom-coated plate. Antibodies have only two binding sites per molecule, and these are on the end on a chain, more susceptible to acid attack. Antigens have several epitopes, which, especially if linear, may be unaffected by hydrolysis elsewhere in the molecule.

An important limitation of the study was that RVV was not detectable in 7 out of 20 patient samples in series 1, which is most likely due to the concentration of RVV being below the limit of detection exacerbated by loss of venom during dissociation treatment. The reason for venom not being detected pre-antivenom (*i.e.*, <2 ng/mL) initially and then post-antivenom is best explained by a smaller delivered venom dose which is then slowly absorbed into the systemic circulation. These few negative cases after dissociation treatment need further investigation, in particular an improved understanding of the absorption kinetics of viper venoms. In addition, a larger study of patient samples needs to be undertaken to fully define the sensitivity and specificity of the assay.

Figure 1 and Figure 2 demonstrate that both the venom concentration and the antivenom concentration change the recovery of the venom after venom dissociation. This means that venom measurements after venom dissociation are not quantitative and only determine if there is venom present or not. The low and essentially zero false positive rate supports the accuracy of detecting venom. Figure 4 shows that the venoms concentrations appeared to be increasing after the administration of antivenom, at least for the available samples assayed. There are a number of possible explanations for this including ongoing gradual absorption of venom into the circulation from the bite site or re-distribution of venom from peripheral sites back into the circulation. It may also be simply that there is a changing ratio of venom to antivenom and therefore changes in venom recovery. Further work is required to investigate this phenomenon.

We have previously shown that venom, if detected in a post-antivenom sample, is largely in bound form, as venom-antivenom complexes [9]. However, for none of the patients in series 1 was venom detectable in untreated post-antivenom samples. Subjecting the samples in series 1 to dissociating conditions revealed the presence of venom in 32 of the 39 cases, and in 50 of 85 in series 2. The method was also successful in series 3 of Australian cases, despite the considerably lower venom concentrations after Australian elapid envenoming.

Dissociation of venom-antivenom complexes with dissociation treatment will be useful in all cases where no pre-antivenom sample is available and there is a question as to whether envenomation has actually occurred, or where the identity of the snake needs to be confirmed. This will be important for both clinical studies, confirmation of envenomation in patients and in forensic medicine.

## 4. Materials and Methods

### 4.1. Materials

Indian polyvalent snake antivenom was obtained from VINS Bioproducts Limited (Hyderabad, Andra Pradesh, India; Batch #01011/10-11). RVV was milked from snakes in Sri Lanka, pooled and then lypholised. Brown snake antivenom was obtained from CSL Ltd (Parkville, Australia, Batch #0559-11001; Expiry 05/13). Stock solutions of venom were prepared as 1 or 2 mg/mL in 50% glycerol and stored at −20 °C. Standard human serum (S7023) and tetramethylbenzidine (TMB) were purchased from Sigma (St Louis, MO, USA), Bovine serum albumin (BSA) from Bovogen (Keilor, Victoria, Australia), and Streptavidin-conjugated horseradish peroxidase (Streptavidin HRP) from Calbiochem (San Diego, CA, USA) (Cat#: OR03L). Blocking solution was 0.5% BSA in phosphate buffered saline (PBS). Washing solution was 0.02% TWEEN 20 in PBS. Polyclonal monovalent rabbit IgG to RVV was obtained by injection of rabbits with RVV followed by purification of the serum on a Protein G-Sepharose column and was carried out at the Western Australian Institute of Medical Research. Rabbit IgG antibodies were biotinylated using EZ-Link Sulfo-NHS-LC-Biotin (Pierce #21335).

### 4.2. Patients

Patients were recruited from three different prospective cohort studies, two studies of Russell’s viper bites in Sri Lanka and one cohort of Australian snake bites. Approval for the collection of the blood samples in Sri Lanka was obtained from Human Research Ethics Committees of the University of Peradeniya (2012/EC/63; approved 2012), University of Rajarata (ERC 2013/019; approved 2013), Monash University (CF14/970–2014000404; approved 2014), University of New South Wales (HERC 10023; approved 2011) and the University of Newcastle (H-2010-1060; approved 2010). Approval for the collection of blood samples in Australia was obtained from Human Research Ethics Committees of the University of Newcastle (H-319-0502; approved 2003), Hunter New England Area Health Service (07/11/21/3.06; approved 2006) and Menzies School of Health Research (HR 03-802; approved 2004). Series 1 was a large cohort of snake-bite patients from two Sri Lankan hospitals [5,18], in which four groups of patients were selected to test the dissociative treatment. The first group was nine definite Russell’s viper bites in which RVV was detected in pre-antivenom samples (positive controls). The second group was 104 non-envenomed patients (no RVV detected) with no coagulopathy or clinical features of systemic envenomation (negative controls). The third group was 30 suspected Russell’s viper bites with coagulopathy that had either no pre-antivenom samples (10) or no venom detected in pre-antivenom samples (20). Coagulopathy or venom induced consumption coagulopathy was diagnosed based on increased prothrombin time (PT), increased activated partial thromboplastin time (aPTT), elevated D-Dimer and low fibrinogen concentrations.

Series 2 of snake bite patients was from a large study of neurotoxicity in Russell’s viper bites [10]. In this study there were a group of patients where Russell’s viper envenomation was suspected but no snake was collected for identification and either venom was not detected pre-antivenom or there was no pre-antivenom sample. Post-antivenom samples were tested after dissociation in 85 suspected Russell’s viper bites.

Patients in Series 3 were selected from patients recruited to the Australian Snakebite Project to investigate the use of the dissociative treatment in other snakes, including taipan (10; *Oxyuranus scutellatus*), brown snake (2; *Pseudonaja textilis*), mulga snake (4; *Pseudechis australis*) and tiger snake (2; *Notechis scutatus*) [7,19,20]. The cases included both positive controls where a pre-antivenom sample was positive for the snake venom and suspected cases in which no pre-antivenom sample was available.

### 4.3. Venom Antivenom Dissociation

Solutions of RVV in standard serum were prepared, then diluted with equal volumes of Indian antivenom in water or with water only. The resulting mixtures had RVV concentrations of 1000 ng/mL, 500 ng/mL, 250 ng/mL, 125 ng/mL and 0 ng/mL, with or without antivenom at 10 mg/mL. These mixtures were allowed to stand at room temperature for an hour then at 4 °C overnight. For venom-antivenom dissociation, 50 µL of the mixture was removed and added to 50 µL of 0.1 M glycine-HCl buffer (pH 2.2) in a microplate. The plate was covered and placed in an oven at 95 °C for 30 min. As a control, a second 50 µL of the venom-antivenom mixture was added to 50 µL of water and not heated (*i.e*., no dissociation treatment). To each well was then added 200 µL of blocking solution, then a portion was removed and further diluted in blocking solution to give a total dilution of 1:120 in the sample. This dilution was applied to a microplate to be assayed for venom, and then further diluted to 1:3120 to measure antivenom.

Solutions of brown snake venom in blocking solution (50 ng/mL) were mixed with increasing concentrations of brown snake antivenom (0 to 2500 mU/mL) and subjected to the dissociation treatment, similar to RVV. Dilutions of 1:14 were then applied to the microplates and assayed for venom using brown snake venom EIA. All dilutions were such that the maximum capacity of the assay was not exceeded (10 ng/mL in well). Dilutions were much greater for the higher venom concentrations seen with RVV, compared to the lower venom concentrations with Australian elapids.

### 4.4. Dissociation of Venom and Antivenom in Patient Samples

Thawed serum samples from patients were mixed with an equal volume of 0.1 M glycine-HCl buffer (pH 2.2) in a microtitre plate. The plate was covered and placed in an oven at 95 °C for 30 min. In a control series water was used instead of 0.1 M glycine-HCl buffer (pH 2.2) and the mixtures were not heated. Both treated and untreated samples were diluted 80 times in blocking solution before being assayed for RVV using the EIA. Samples from Australian snake bite patients were subject to the same conditions except were measured at less dilution. Again, sample dilutions were based on known venom concentrations which are much higher for RVV. In cases where RVV was not detected after venom dissociation, samples were re-run at a dilution of 1:10 to increase sensitivity.

### 4.5. Venom Enzyme Immunoassay

RVV was detected using a sandwich EIA as previously described [5,7,20] in thawed samples from the three series of patients. In brief, Greiner Microlon 96-well high-binding plates were coated with 100 µL of rabbit anti-RVV IgG (1 µg/mL) in carbonate buffer (50 mM, pH 9.6), kept at room temperature for 1 h and then at 4 °C overnight. The plates were then washed and 300 µL of blocking solution was applied. After 1 h the plates were washed again, and 100 µL of sample solution was applied. The plates were allowed to stand for 1 h and then washed three times. Next, 100 µL of biotinylated anti-RVV IgG (0.3 µg/mL in blocking solution) was added. After standing for a further hour the plates were washed again. Streptavidin-horseradish peroxidase (100 µL, 0.1 µg/mL in blocking solution) was added and left for 1 h. The plate was then washed three times and 100 µL of TMB reagent added and colour allowed to develop for 3.5 min. The reaction was stopped by the addition of 50 µL of 1 M H_2_SO_4_. Plates were read on a BioTek Synergy HT instrument (BioTek Instruments Inc, Winooski, VT, USA) at 450 nm. All samples were measured in triplicate, and the averaged absorbance converted to a venom concentration by comparison with a standard curve based on eight serial dilutions of venom from 10 ng/mL to 0 ng/mL in blocking solution (concentration in the well). Data was fitted to a sigmoidal dose-response curve using GraphPad Prism software version 6.03 for Windows, GraphPad Software (San Diego, CA, USA).

Taipan, brown snake, mulga snake and tiger snake venoms were detected using a sandwich EIA, as previously described [7] and similar to RVV. For the Australian snake bite cases when venom from the suspected snake type was not detected, further snake venoms were then assayed based on the next most likely snakes to be involved considering geography, and clinical and laboratory effects.
